# Amygdala subnuclei development in adolescents with autism spectrum disorder: Association with social communication and repetitive behaviors

**DOI:** 10.1002/brb3.2299

**Published:** 2021-08-01

**Authors:** Diane Seguin, Sara Pac, Jianan Wang, Rob Nicolson, Julio Martinez‐Trujillo, Emma G. Duerden

**Affiliations:** ^1^ Physiology and Pharmacology Schulich School of Medicine and Dentistry, Western University London Canada; ^2^ Neuroscience Schulich School of Medicine and Dentistry, Western University London Canada; ^3^ Biomedical Engineering Faculty of Engineering, Western University London Canada; ^4^ Psychiatry, Schulich School of Medicine and Dentistry University of Western Ontario London Canada; ^5^ Applied Psychology Faculty of Education, Western University London Canada

**Keywords:** amygdala, autism spectrum disorders, behavior, human, magnetic resonance imaging, social

## Abstract

**Introduction:**

The amygdala subnuclei regulate emotional processing and are widely implicated in social cognitive impairments often seen in children with autism spectrum disorder (ASD). Dysregulated amygdala development has been reported in young children with ASD; less is known about amygdala maturation in later adolescence, a sensitive window for social skill development.

**Methods:**

The macrostructural development of the amygdala subnuclei was assessed at two time points in a longitudinal magnetic resonance imaging (MRI) study of adolescents with ASD (*n* = 23) and typically‐developing adolescents (*n* = 15) . In adolescents with ASD, amygdala subnuclei growth was assessed in relation to ASD symptomatology based on standardized diagnostic assessments. Participants were scanned with MRI at median age of 12 years and returned for a second scan at a median age of 15 years. The volumes of nine amygdala subnuclei were extracted using an automatic segmentation algorithm.

**Results:**

When examining the longitudinal data acquired across two time points, adolescents with ASD had larger basolateral amygdala (BLA) nuclei volumes compared to typically developing adolescents (*B* = 46.8, *p* = 0.04). When examining ASD symptomatology in relation to the growth of the amygdala subnuclei, reciprocal social interaction scores on the ADI‐R were positively associated with increased growth of the BLA nuclei (*B* = 8.3, *p* < 0.001). Growth in the medial nucleus negatively predicted the communication (*B* = −46.9, *p* = 0.02) and social (*B* = −47.7, *p* < 0.001) domains on the ADOS‐G. Growth in the right cortical nucleus (*B* = 26.14, *p* = 0.02) positively predicted ADOS‐G social scores. Central nucleus maturation (*B* = 29.9, *p* = 0.02) was associated with the repetitive behaviors domain on the ADOS‐G.

**Conclusions:**

Larger BLA volumes in adolescents with ASD may reflect underlying alterations in cellular density previously reported in post‐mortem studies. Furthermore, findings demonstrate an association between regional growth in amygdala subnuclei volumes and ASD symptomatology. Improved understanding of the developmental trajectories of the amygdala subnuclei may aid in identifying key windows for interventions, particularly for social communication, in adolescents with ASD.

## Graphical abstract

Amygdala subnuclei maturation was examined in adolescents with and without ASD. The volumes of the BLA nuclei were larger in adolescents with ASD compared to controls. BLA, cortical, and medial nuclei predicted social and/or communication skills. Larger central nuclei volumes positively predicted repetitive behaviors.

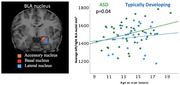

## 1 INTRODUCTION

Autism spectrum disorder (ASD) is a neurodevelopmental disorder characterized by atypicalities in social behaviors and communication, as well as repetitive movements, and restricted interests (American Psychiatric Association 2013). These behavioral findings have led to functional and anatomical imaging studies to uncover the neurological underpinnings of ASD symptomatology (Dekhil et al., 2020; Philip 2012; Watanabe & Rees 2016). Given the role of the amygdala and other limbic structures in social interaction, they have become strong targets for research in individuals with ASD relative to neurotypically developed controls (Zalla & Sperduti 2013).

The amygdala is a bilateral subcortical structure located in the medial temporal lobes and composed of several nuclei. These nuclei can be individually segmented from high‐field MR images using automated segmentation techniques (Saygin et al., 2017) and from cell staining post‐mortem tissue samples (Schumann & Amaral 2006). Amygdala subnuclei differ in their cytoarchitecture and receive inputs from, and project to, frontal and temporal cortical areas (LeDoux 2007). Given the known role of the amygdala in sensory and emotional processing, research has focused on the unique contributions of the amygdala subnuclei in typical and atypical amygdala functioning. The basolateral amygdala (BLA) complex is the main receiver of sensory information (deCampo & Fudge 2012).

The amygdala is responsible for emotional processes as well as fear, learning, and memory. The amygdala undergoes rapid changes in size during infancy (Uematsu et al., 2012). Development of the amygdala is nonlinear, increasing in size during childhood and adolescence, then undergoing slight decreases in total size around late adolescence and early adulthood (Ostby et al., 2009; Wierenga et al., 2014; Wierenga et al., 2018). The developmental stages of the amygdala correspond with social and emotional growth and maturity that occur as children age (Qin et al., 2012).

Studies of infants have reported an overgrowth of the amygdala in children later diagnosed with ASD (Avino et al., 2018; Schumann et al., 2009), with some evidence that this increased growth begins around 6 months of age (Li et al., 2019). In a small post‐mortem study, fewer neurons were found in lateral nuclei of males with ASD compared to neurotypical males (Schumann & Amaral 2006). Increased amygdala volumes have also been found in adolescents with ASD (Groen et al., 2010). In TD children, amygdala growth plateaus during adolescence (Ostby et al., 2009). Other studies have reported equivalent amygdala volumes in both ASD and TD adults and older teens (Schuman et al., 2004; Xu et al., 2020). Neuronal counts have revealed differences in neuronal growth trajectories across the lifespan between individuals with ASD and those who are neurotypical (Avino et al., 2018). Recent studies report whole amygdala growth during childhood in TD children, with delayed growth during adolescence (Schumann et al., 2004; Xu et al., 2020). Altogether, evidence suggests alterations in the growth of the amygdala in children with ASD compared to TD children. Given the complex organization of the amygdala subnuclei, more detailed analyses are needed to determine whether delayed growth is evident throughout the entire amygdala, or whether there are different growth patterns seen across amygdala subnuclei in adolescents with ASD.

Studies have supported the theory that enlargements of the amygdala in children are associated with more severe social and communication impairments (Kim et al., 2010; Lucibello et al., 2019; Munson et al., 2006). Conversely, smaller amygdala volumes in adult males with ASD were associated with poorer emotional recognition and joint attention in their childhood (Nacewicz et al., 2006). More severe social impairments were associated with increased amygdala size in a recent meta‐analysis of ASD and neurotypical subjects across the lifespan (van Rooij et al., 2018) while a large cohort study of children with neurodevelopmental disorders found a similar association between amygdala volume and socio‐emotional functioning (Baribeau et al., 2019). Differences in amygdala morphometry have been implicated in other studies of pediatric pathologies that share some behavioral phenotypes seen in children with ASD, including generalized anxiety disorder (De Bellis et al., 2000; Warnell et al., 2018), fearfulness (van der Plas et al., 2010), attention deficit/hyperactivity disorder, and obsessive–compulsive disorder (Baribeau et al., 2019). With such overlap in behaviors exhibited amongst children ASD and these other disorders, it was hypothesized that an association between amygdala subnuclei and ASD symptomatology would be evident in adolescents with ASD.

The BLA complex is the largest of the amygdala subnuclei and is the main reception site for sensory information (deCampo & Fudge 2012). The BLA receives multi‐sensory information from the thalamus, hippocampus, and cortex and in turn projects to other amygdala nuclei, as well as the orbital frontal cortex (OFC), dorsal and ventral striatum, and the anterior cingulate cortex, among other brain regions (Davis & Whalen 2001). The BLA has been theorized to function as a “gate‐keeper” by assessing incoming sensory information and assigning emotional saliency to appropriate stimuli before the information is sent to other brain regions. Alterations to the BLA may affect downstream systems involved in social cognition and decision‐making, such as the OFC and mPFC, respectively (Sinha et al., 2015).

The central (Ce) nucleus transmits information received from the BLA to both the hypothalamus and the regions of the brainstem which are responsible for carrying fear responses (LeDoux et al., 1988). Neurons in the Ce nucleus express high numbers of oxytocin receptors (Huber et al., 2005) and it is believed that oxytocin activation in these neurons mediates fear responses. Increased oxytocin has been shown to reduce activity of the Ce nucleus, which in turn resulted in decreased anxiety behaviors in an animal model (Kirsch et al., 2005).

Animal studies have reported the importance of the medial amygdala nuclei in influencing both autonomic and behavioral responses to environmental stimuli (Vinkers et al., 2010). During typical development, the medial nucleus of the amygdala is responsible for mediating social behaviors including social recognition and same‐sex rivalry (Haller 2018; Wang et al., 2014).

Both the cortical amygdala nucleus (CoA) and the anterior amygdaloid area (AAA) receive olfactory information in rodents (Cadiz‐Moretti et al., 2016). The CoA receives pheromonal information in rodents and is thought to contribute to memory processes (Kempipainen & Jolkkonen 2002). The AAA receives input from both the main and accessory olfactory bulbs in rodents (Cadiz‐Moretti et al., 2017). Both the CoA and the AAA are likely to be involved in defensive behaviors, and in fear conditioning. Although underreported on in human studies, some recent evidence has shown the AAA, and other amygdala subnuclei, to be smaller in adults with major depressive disorder (Yao et al., 2020) and those diagnosed with schizophrenia (Zheng et al., 2019). Similarly, the corticoamygdaloid transition area (CAT) nuclei was found to be significantly smaller in patients diagnosed with schizophrenia or bipolar disorder (Barth et al., 2021). The CTA has been implicated in processing negative facial expressions (Kilts et al., 2003). Due to their roles in olfactory and facial processing, the CoA, AAA, and CAT are believed to be important contributors to social communication (Bzdok et al., 2013).

The paralaminar nucleus (PL) is an understudied nucleus of the amygdala, in part as it is significantly proportionally smaller and more difficult to discern in rodent brains than in humans (Decampo & Fudge, 2012). This nucleus is known to contain large numbers of immature neurons, which persist well into adulthood (Sorrell et al., 2019). Recently, smaller PL volumes were associated with posttraumatic stress disorder (PTSD) prevalence in military veterans, with the authors speculating that the neuroplasticity of this particular nucleus may result in its increased susceptibility to the damaging effects of trauma (Morey et al., 2021).

The association between restrictive and repetitive behaviors and the amygdala has not been as extensively studied as has the relationship between the amygdala and social impairments associated with ASD. Overall, larger amygdala volumes are associated with ASD diagnosis in young children (Nordahl et al., 2012), with one study reporting a significant relationship between larger total amygdala volumes in toddlers with ASD and increased repetitive behaviors (Breece et al., 2013). In contrast, smaller amygdala volumes are exhibited by adult patients with schizophrenia (Zhang et al., 2020) and OCD (Zheng et al., 2019), disorders associated with increased motor behaviors and patterns. Further investigation is needed to determine whehter amygdala subnuclei display a relationship with restrictive and repetitive behaviors in ASD.

The amygdala nuclei are involved in diverse functions and have been implicated in various disorders, particularly those which involve mood or anxiety dysfunction (LeDoux, 2007). To our knowledge no study has yet investigated amygdala subnuclei differences during adolescent development in youths with ASD. Given the heterogeneity of symptoms displayed in youth with ASD longitudinal studies which examine changes in developing amygdala nuclei morphology are necessary to fully understand how the development of the amygdala is associated with social–emotional processing in children and adolescents. Despite that, few studies to date have examined longitudinal maturational trajectory of the amygdala in children and adolescents with ASD. As well, further studies are needed to examine the amygdala subnuclei volumes that are associated with ASD symptom severity.

Of particular interest to our study is ASD symptomatology that is assessed by the Autism Diagnostic Interview, Revised (ADI‐R) (Lord et al., 1994), and Autism Diagnostic Observation Schedule‐G (ADOS‐G) (Lord et al., 2000), both are widely used gold‐standard diagnostic tests. These tests assess for behaviors such as restrictive and repetitive behaviors that may be motor movements (stereotypies), which may include body rocking and hand flapping or ways of interacting with objects, such as playing with toys in a particular order. Atypical social behaviors may include reduced or absent eye contact and joint attention, difficulties with social reciprocity, and engaging in social play activities. Communication differences commonly seen in children with ASD include limited or absent vocal speech, limited nonverbal communication, and limited responses to verbal and nonverbal cues from others.

Using two longitudinal datasets of adolescents with and without ASD, this study addresses two main questions: Are there differences in the developmental trajectories of the amygdala subnuclei between adolescents with ASD and typically developing adolescents? Does the macrostructural growth of amygdala subnuclei predict ASD symptom severity?

To address our first question the amygdala subnuclei volumes at each time point were examined in relation to group (ASD vs. typically developing, TD), adjusting for demographic variables. We predicted that adolescents with ASD would demonstrate larger amygdala subnuclei volumes compared to TD children at both time points. To address our second question, the growth of the amygdala subnuclei were examined in relation to standardized tests of ASD symptomatology, which included measures of social cognition, communication, and restricted and repetitive behaviors. We tested two predictions:( 1) subnuclei volume maturation would positively predict social cognition and communication scores; (2) restricted and repetitive behaviors in children with ASD would be associated with variations in the patterns of growth in the subnuclei.

## 2 METHODS

### 2.1 Participants

Adolescents with ASD and typically developing (TD) children were tested at two sites: University of Pittsburgh Autism Center of Excellence (ACE) subject core and University of California Los Angeles' (UCLA) Center for Autism Research and Treatment (CART). Participants were scanned at a median age of 12 years and returned for a second scan 1 to 4 years later. The data were made available through the ABIDE II project (http://fcon_1000.projects.nitrc.org/indi/abide/abide_II.html).

At both sites, participants with ASD were diagnosed using the ADI‐R (Lord et al., 1994) and ADOS‐G (Lord et al., 2000) at both study time points. The ADI‐R is a semi‐structured interview conducted with parents or caregivers on the behavioral development of the child in relation to ASD symptomatology, while the ADOS‐G consists of a clinician's assessment of the child's behavior across multiple activities. All children with ASD were assessed with the ADOS‐G module 3 except for three children who were assessed with module 4. Exclusion criteria for TD adolescents included neurodevelopmental disorders, seizures, learning disabilities, and psychiatric disorders and were based on parent report. The research ethics boards at the respective institutions approved the study. Parents provided informed consent and children provided assent to participate in the study.

### 2.2 Psychological testing

At both sites, adolescents underwent psychological testing by trained psychometrists. All children were assessed on the Wechsler Abbreviated Scale of Intelligence (WASI) (Wechsler, 1999) or the Wechsler Intelligence Scale for Children‐Fourth Edition (WISC‐IV) (Wechsler, 2014).

### 2.3 Magnetic resonance imaging

At UCLA anatomical scans were acquired on a Siemens Tim Trio with a 12‐channel head coil using a three‐dimensional T1‐weighted MPRAGE sequence (field of view [FOV] = 256 × 240 × 192, 1 × 1 × 1.2 mm voxels, repetition time [TR] = 2300, inversion time [TI] = 900 ms, echo time[TE] = 2.86 ms, flip angle = 9°).

Children recruited from the University of Pittsburgh were imaged at the Neuroscience Imaging Center at the McGowan Institute for Regenerative Medicine at the University of Pittsburgh on a Siemens Allegra 3T MRI scanner. Using an 8‐channel head coil, a magnetization‐prepared rapid gradient echo (MPRAGE) was acquired in all participants (FOV = 256 × 240 × 192 mm, 1.1 × 1.1 × 1.05 mm voxels, TR = 2100, TI = 1000, TE = 3.93 ms, flip angle = 7°).

In order to adjust for differences in scanner type as well as the scanning protocols, all analyses were corrected for study site.

### 2.4 Cortical and subcortical segmentation

The total cerebral volume, whole amygdala, and nine of the subnuclei were segmented automatically using FreeSurfer (http://surfer.nmr.mgh.harvard.edu) version 6.0. The amygdala segmentation algorithm is available through the development version. The algorithm is based on Bayesian inference, and the amygdala atlas was developed using ex vivo human sections of the medial temporal lobes (*n* = 10).

In the current study, the amygdala was segmented into the central (Ce), lateral, basal, accessory basal, cortical, medial, and paralaminar nuclei as well as the CTA and the AAA. The nuclei forming the BLA complex (basal, lateral, and accessory basal nuclei) were summed and were used as predictor variables in the subsequent analyses.

Protocols for quality control included visual inspection of the postprocessed amygdala segmentations using the Freeview image viewing platform, within the FreeSurfer package. Postprocessed images were visually inspected to determine the accuracy of the algorithm used to segment the nuclei using anatomical atlases as reference material (Amunts et al., 2005; Solano‐Castiella et al., 2011). The volumes of the subnuclei were automatically extracted using an in‐house developed software, which is available on the Developing Brain Lab GitHub site: https://github.com/DevelopingBrainLab/amygdala_segmentation_Freesurfer

### 2.5 Statistical analysis

Statistical analyses were carried out using SPSS (version 26, Statistical Package for the Social Sciences, IBM, Chicago, IL). In a first step, in a series of generalized estimating equations models we examined the averaged left and right volumes of the amygdala subnuclei (Ce, BLA, cortical, medial, and paralaminar nuclei, and the CTA and AAA; dependent variables) at each time point in relation to diagnostic group (ASD, typically developing; independent variables), taking into account within‐subject factors (site, participant id, scan) and adjusting for sex, age, IQ, and total cerebral volumes. As part of our analytic plan to examine laterality differences, the analyses were repeated using the left and right subnuclei volumes separately that differed significantly between diagnostic groups.

To examine the association of the macrostructural growth of the amygdala subnuclei with ASD symptomatology, we first calculated the weekly growth of the amygdala subnuclei in cubic millimeters. We subtracted the volumes at time point 1 from the volumes at time point 2 and divided by the difference in the ages at the time of the scan (i.e., [volume [cubic millimeters] time 2/age [weeks] scan 2] – [volume time 1/age scan 1]) using a series of generalized linear models (GLM), with an identity link function, we examined the total scores for the ADI‐R and the ADOS‐G subscales in relation to the weekly growth in cubic millimeters of amygdala subnuclei (e.g., ADOS‐G Social ∼ 1 + weekly growth mm^3^ + site + sex + age + IQ).

As we had one a priori hypothesis regarding the association of ASD diagnosis and amygdala subnuclear volumes, the alpha level for statistical tests was set at *p* = 0.05. We had two predictions in relation to the outcome scores for social communication abilities and repetitive behaviors, assessing the volumes and the association with the scores on the ADI‐R and the ADOS‐G, the alpha level was set at *p* = 0.025.

## 3 RESULTS

### 3.1 Participant demographics

A total of 23 children with ASD and 15 typically developing adolescents participated in the study. Nine of the participants with ASD and 8 of the typically developing adolescents were recruited through the University of Pittsburgh's ACE subject core. Fourteen children with ASD and 7 of the typically developing adolescents were recruited through recruited through UCLA's CART. The participant characteristics are listed in Table 1. The median age of the all participants (ASD and TD) at scan 1 was 12.6 years (interquartile range [IQR]: 11.5–13.8). The participants returned for a second scan at a median age of 15.1 years (IQR:13.9–16.1). The ADOS‐G and ADI‐R scores were available for 22 children with ASD (Table 2).

**TABLE 1:** Participant characteristics

	TD	ASD
*n*	15	23
Age scan 1 [IQR]	12.9 [12–14.2]	12.2 [11.2–13.2]
Age scan 2 [IQR]	15.9 [14.5–16.5]	14.6 [13.5–15.5]
Males (%)	13 (87)	20 (87)
Right handed (%)	14 (93)	20 (87)
*IQ [IQR]*†		
VIQ	109 [100–115]	104 [89–111]
PIQ	116 [108–119]	98 [89–116]
FSIQ	112 [107–121]	103 [88–117]

†All typically developing children (n = 15) and 11 children with ASD completed the Wechsler Abbreviated Scale of Intelligence (WASI), and the remaining 12 children completed the Wechsler Intelligence Scale for Children, fourth edition (WISC‐IV).

**TABLE 2:** Autism diagnostic scales

*ADOS‐G*	
*n*	22
Total	12.5 (12–14)
*Domains*	
Nonverbal and verbal communication	4 (3–5)
Reciprocal social interaction	9 (8–10)
Repetitive and stereotyped behaviors	3 (2–3)
*ADI‐R*	
*n*	22
Reciprocal social interaction domain	5 (16–23)
Nonverbal and verbal communication domain	15 (11–19)
Repetitive and stereotyped behavior domain	5 (4–6)

Abbreviations: ADOS‐G, Autism Diagnostic Observation Scale, General; ADI‐R, Autism Diagnostic Interview, Revised.

### 3.2 Neuropsychological testing

All typically developing children (*n* = 15, 100%) and 11 (48%) children with ASD completed the WASI, and the remaining 12 (52%) children with ASD completed the WISC‐IV (Table 1).

### 3.3 Volumetric maturation of the amygdala subnuclei

**FIGURE 1:**
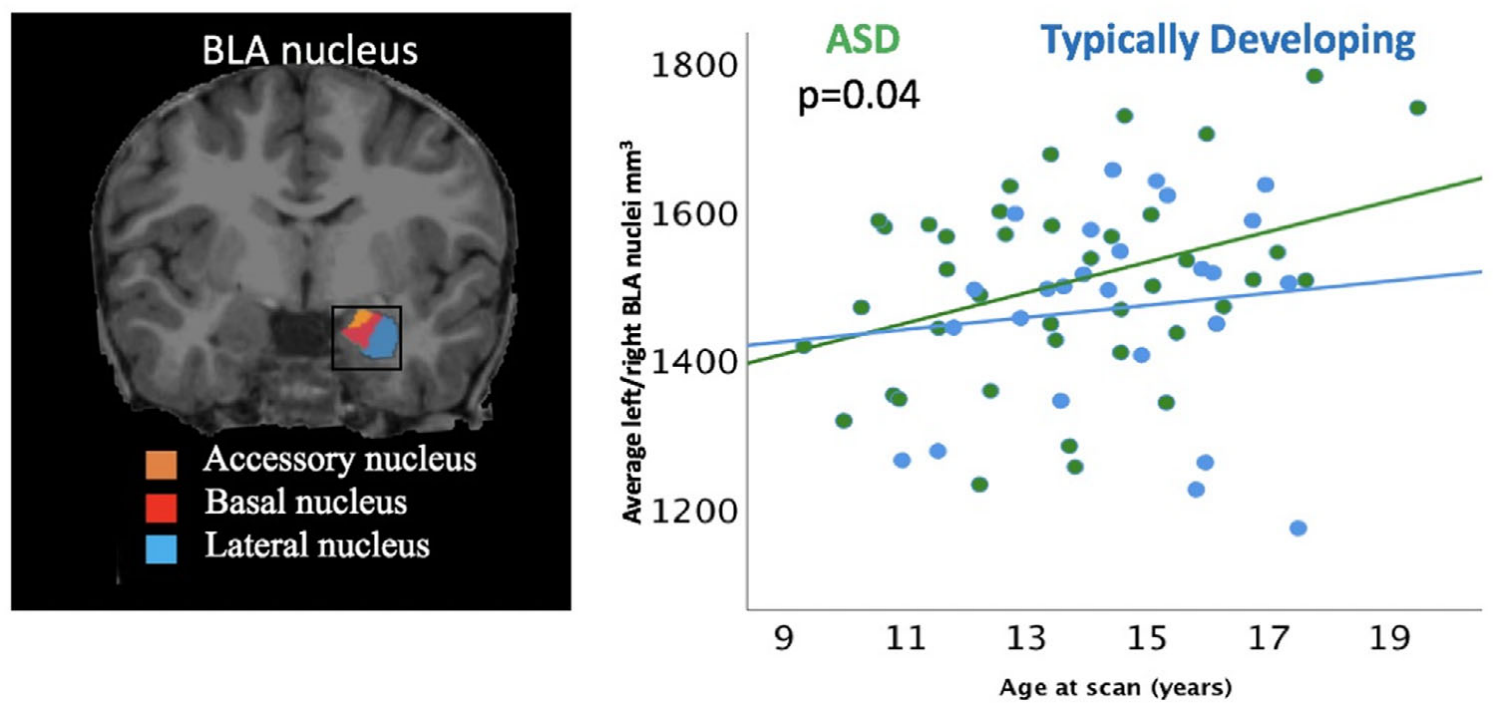
The volumes of BLA nuclei (cubic millimeters [mm^3^], *y* axis) from both time points examined in relation to ASD diagnosis (ASD—green; TD—blue) and age (years, *x* axis)

In the first step, we examined the association of the volumes of the subnuclei, acquired at both time points, in relation to diagnosis, in a series of generalized estimating equations, adjusting for age at scan, full scale IQ and total cerebral volumes. Across the two time points, the average BLA nuclei volumes were significantly different between groups, with children with ASD demonstrating larger volumes relative to typically developing children (*B* = 46.8, *p* = 0.04, Figure 1). Age (*B* = 9.4, *p* = 0.009), male sex (*B* = 93.1, *p* = 0.01) and total cerebral volumes (*B* = 0.001, *p* < 0.001) were significant in the model. Results were maintained when adjusting for medication usage (*B* = 47.8, *p* = 0.03). No significant volumetric differences between participant groups were evident for any other amygdala subnuclei (*p* > 0.05).

**FIGURE 2:**
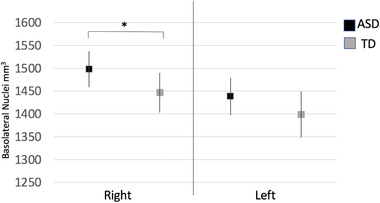
Volumes of left and right BLA nuclei (cubic millimeters [mm^3^], *y* axis) from both time points examined in relation to diagnosis (ASD—black; TD—grey). Values represent the estimated marginal means of the volumes of the BLA, adjusting for site, scan, sex, age, IQ, and total cerebral volumes based on the output from the generalized estimating equation model. The right BLA subnuclei were significantly larger in adolescents with ASD (*p* = 0.02). *<0.05

We subsequently performed an analysis to examine whether the effects in the BLA were lateralized to one hemisphere. We repeated the analysis using the volumes of the BLA from the left and right and hemispheres in two separate analyses. The right BLA demonstrated significant between group differences (*B* = 52.7, *p* = 0.02), with adolescents with ASD demonstrating larger volumes in relation to TD adolescents (Figure 2). Age, male sex, and total cerebral volumes were significant positive predictors of right BLA volumes (all, *p* < 0.04). Results were similar when adjusting for medication use (*B* = 54.9, *p* = 0.02). We then performed a confirmatory analysis to examine the volumetric intercorrelations amongst the amygdala subnuclei. All nuclei volumes were positively correlated with one another (all, *p* < 0.05) with the exception of the paralaminar and medial subnuclei, which showed positive but nonsignificant associations (*r* = 0.2, *p* = 0.1).

### 3.4 Maturation of the amygdala subnuclei and association with ASD symptomatology

We subsequently examined the macrostructural weekly growth (in cubic mm) of the amygdala subnuclei and the association with the subtests of the ADI‐R (1. Language/communication, 2. Reciprocal social interactions, and 3. Restricted, repetitive, and stereotyped behaviors and interests) and ADOS‐G (1. Social interactions, 2. Verbal and nonverbal communication, 3. Stereotyped behaviors and restricted interests) in a series of GLMs.

The total reciprocal social interactions score from the ADI‐R was predicted by the weekly growth in the BLA nuclei (*B* = 8.3, *p* < 0.001, Table 3), adjusting for site and biological sex. As part of an exploratory analysis, we examined the association of the growth of the left and right BLA nuclei with reciprocal social interactions assessed using the ADI‐R in a GLM, adjusting for the same covariates as in the original models. Results indicated that neither the left or right BLA showed an association with the reciprocal social interaction score on the ADI‐R (both, *p* > 0.3). None of the other subtests of the ADI‐R were predicted by growth of the amygdala subnuclei (both, *p* > 0.025).

**TABLE 3:** Results of a general linear model demonstrating the association among the ADI‐R social score and volumes of the subnuclei of the amygdala in children with ASD

		95% Wald confidence interval		
	Beta	Lower	Upper	*p* value
Male sex	**−7.1**	**−10.4**	**−3.8**	**<0.001**
Site	3.0	−0.6	6.6	0.1
AAA nucleus growth	16.6	−45.0	78.2	0.6
Central nucleus growth	16.5	−25.9	58.8	0.45
Medial nucleus growth	−47.4	−124.3	29.5	0.23
Cortical nucleus growth	77.5	−79.5	234.5	0.33
CTA nucleus growth	−2.4	−27.3	22.6	0.85
PL nucleus growth	−63.7	−160.7	33.3	0.2
BLA nucleus growth	**8.3**	**3.46**	**13.24**	**<0.001**

Abbreviations: AAA, anterior amygdaloid area; CTA, corticoamygdaloid transition area; PL, paralaminar nucleus; BLA, basolateral nucleus

**FIGURE 3:**
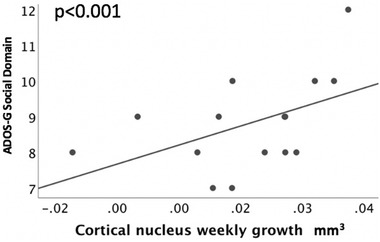
ADOS‐G social interaction scores (ADOS‐G Social Domain) in adolescents with ASD in relation to the weekly growth of the cortical nucleus (mm^3^)

**FIGURE 4:**
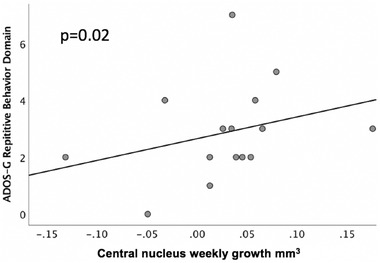
ADOS‐G repetitive behavior domain scores in adolescents with ASD in relation to the weekly growth of the central (Ce) nucleus (mm^3^)

When examining the subdomains of the ADOS‐G in relation to the growth of the amygdala subnuclei, the communication subscale on the ADOS‐G was negatively predicted by the growth in the medial nucleus (*B* = −46.9, *p* = 0.02, Table 4.). Additionally, the social domain on the ADOS‐G was negatively predicted by growth in the medial nucleus (*B* = −47.7, *p* < 0.001, Table 5) and positively by growth in the cortical nucleus (*B* = 102.1, *p* < 0.001, Figure 3). We subsequently examined laterality differences in the cortical nucleus, and only the right‐sided volumes (*B* = 26.14, *p* = 0.02) positively predicted ADOS‐G social scores. The repetitive behavior domain on the ADOS‐G was positively predicted by the growth in the Ce nucleus (*B* = 29.9, *p* = 0.02, Figure 4).

**TABLE 4:** Results of a general linear model demonstrating the association among the ADOS‐G communication subscale and volumes of the subnuclei of the amygdala in children with ASD

		95% Wald confidence interval		
	Beta	Lower	Upper	*p* value
Male sex	−1.2	−2.8	0.5	0.17
Site	0.6	−1.3	2.4	0.55
AAA nucleus growth	14.0	−17.6	45.6	0.39
Central nucleus growth	14.4	−7.3	36.1	0.19
Medial nucleus growth	**−46.9**	**−86.4**	**−7.5**	**0.02**
Cortical nucleus growth	52.8	−27.8	133.3	0.20
CTA nucleus growth	9.6	−3.2	22.4	0.14
PL nucleus growth	−24.9	−74.6	24.9	0.33
BLA nucleus growth	1.1	−1.4	3.6	0.37

Abbreviations: AAA, anterior amygdaloid area; CTA, corticoamygdaloid transition area; PL, paralaminar nucleus; BLA, basolateral nucleus.

**TABLE 5:** Results of a general linear model demonstrating the association among the ADOS‐G social subscale and volumes of the subnuclei of the amygdala in children with ASD

		95% Wald confidence interval		
	Beta	Lower	Upper	p value
Male sex	0.73	−0.2	1.7	0.14
Site	0.74	−0.3	1.8	0.18
AAA nucleus growth	−6.81	−25.2	11.6	0.47
Central nucleus growth	9.01	−3.6	21.7	0.16
Medial nucleus growth	**−47.7**	**−70.7**	**−24.8**	**<0.001**
Cortical nucleus growth	**102.1**	**55.2**	**149.0**	**<0.001**
CTA nucleus growth	7.76	0.3	15.2	0.04
PL nucleus growth	−11.89	−40.9	17.1	0.42
BLA nucleus growth	0.44	−1.0	1.9	0.56

Abbreviations: AAA, anterior amygdaloid area; CTA, corticoamygdaloid transition area; PL, paralaminar nucleus; BLA, basolateral nucleus.

## 4 DISCUSSION

In a longitudinal cohort study of children and adolescents with and without ASD, the macrostructural growth of the amygdala subnuclei was examined using automatic segmentation methods to address two main questions: (1) Do developmental trajectories of the subnuclei differ between adolescents with ASD and typically developing adolescents?; (2) Is the growth of the nucleic volumes associated with ASD symptomatology? Larger BLA nuclei were observed in adolescents with ASD compared to their typically developing peers, and these differences were more pronounced at older ages. Additionally, in adolescents with ASD, volumes of the BLA and cortical nuclei were positively associated with impaired social communication, while medial nuclei volumes negatively predicted social and communication impairments. Larger Ce nuclei volumes were associated with increased repetitive behaviors in children with ASD. To better understand the clinical significance of the increases and decreases in volumes and in relation to longer‐term outcomes, particularly social behaviors, future longitudinal studies with larger cohorts at older ages are needed.

The BLA, the main sensory processing unit of the amygdala (LeDoux 2000), was larger in adolescents with ASD compared to their typically developing peers. When examining amygdala nucleic growth across both time points, increased growth of the BLA was predictive of social communication impairments, as measured by the ADI‐R in participants with ASD. The BLA registers emotional salience and emotional states of others through its reciprocal connections to the prefrontal cortex and orbitofrontal cortex (Truitt et al, 2007). Additionally, the BLA contributes to setting anxiety levels during social interactions. Disruption of the inhibitory circuitry of the BLA was associated with reduced anxiety responses during social interactions (Truitt et al., 2007). BLA dysfunction could result in an exaggerated salience processing of social cues, reduced inhibition, and increased anxiety. Adolescents with ASD who struggle with interpreting and responding to social cues may also avoid social interactions as a consequence of exaggerated social fear (Nacewicz et al., 2006; Sinha et al., 2015; Truitt et al., 2007). Deep brain stimulation of the BLA has been proposed as a possible treatment for social anxiety in individuals with ASD (Sinha et al., 2015). The volumetric changes in the BLA, seen in the current study, may also be associated with altered neuronal activation of this nucleic complex in individuals with ASD in response to social stimuli (Rutishauser et al., 2013).

Slower growth of medial subnucleic volumes was associated with greater impairments on both the communication and social domains assessed with the ADOS‐G, while increased growth of cortical nuclei predicted social communication impairments. Both the cortical and medial nuclei are involved in processing olfactory cues necessary for social recognition in mammals (Gutiérrez‐Castellanos et al., 2014; Sah et al., 2003), which underlies social functions such as maternal and sexual behaviors (Meurisse et al., 2009). Our findings suggest both medial and cortical neurons contribute to social functioning similarly in humans. As the medial nucleus is implicated in facilitating environmental responses it may be that alterations in macrostructural development disrupt typical mediation of behavioral responses to social stimuli.

Atypical sensory processing is common in individuals with ASD. As the medial nucleus is involved in processing both environmental and social stimuli (Vinkers et al., 2010; Wang et al., 2014), the smaller volumetric size of this nucleus may reflect excitotoxicity, whereby increased nucleic activity due to increased sensitivity to environmental stimuli leads to increased neuronal death (Avino et al., 2018). In turn, the individual then experiences difficulties in social contexts. It is also possible that the smaller size of this nuclei reflects atypical development of this nuclei, resulting in its smaller volume, and the associated impairments in social and communication behaviors.

In the current work, increased volumetric growth of the Ce nucleus was a positive predictor of repetitive behaviors in children with ASD as measured by the ADOS‐G. The primary function underlying repetitive behaviors is emotional and physiological regulation (Samson et al., 2014). Individuals engage in repetitive stereotypic behaviors possibly as a means of self‐soothing during periods of increased anxiety (Joosten et al., 2009). Children with ASD who display high rates of stereotypy also report higher anxiety than children with ASD who display more mild stereotyped behaviors (Baribeau et al., 2020).

Chronic stress has been shown to reduce dendritic growth in BLA neurons while sparing the Ce nucleus in rodent models (Vyas et al., 2003). The enlarged growth of the Ce nucleus and its association with repetitive behaviors may be indicative of dysfunctional stress responding in these children. Further investigation of other brain regions involved in stress processing including the hippocampus and prefrontal cortex, as well as the associated circuitry, are needed in this population.

Of note, ASD symptomatology assessed by the ADI‐R and ADOS‐G were predicted by the macrostructural growth of different subnuclei, findings that likely reflect methodological differences in the diagnostic tests. The ADI‐R relies on parent/caregiver reports, while the ADOS‐G is a clinician‐administered test in tightly structured social scenarios. Both assessments are commonly used in combination to diagnose children presenting with ASD symptoms to better capture particular behaviors exhibited by each child (Frigaux, et al., 2019). The disparity in our findings, whereby subnuclear growth predicted different domains on the ADOS‐G and ADI‐R, may reflect measurement differences at the different sites or recall biases.

No significant volumetric differences in PL, CTA, or AAA nuclei were evident between groups. Furthermore, we found no relationship between these subnuclei volumes and ASD symptomatology. Human and animal studies report that these subnuclei contribute to processing negative and fearful stimuli, particularly visual stimuli. Small volumes of these nuclei were reported in adults diagnosed with depression (Yao et al., 2020), bipolar disorder (Barth et al., 2021), and schizophrenia (Zheng et al., 2019). It is possible these nuclei are more heavily involved in mood regulation thus they did not provide meaningful contributions to the ASD symptomatology captured in our sample.

We reported relationships between both larger and smaller nuclei with increased ASD symptoms. As smaller nuclei have been reported in other psychiatric disorders such as depression (Yao et al., 2020), schizophrenia (Zheng et al., 2019), and anxiety (Herrington et al., 2017), it is possible that the amygdalae in these affected individuals are more vulnerable to stress, leading to neuronal death and behavioral impairments. Our findings of smaller nuclei volume associated with greater social and communication impairments support this possibility. Further investigation into the growth trajectories of each nuclei across development, and associations with ASD symptoms, are necessary to determine which amygdala functions contribute to these behavioral differences.

In a recent study of amygdala subnuclei growth during healthy cognitive aging, BLA and cortical nuclei volumes were nonlinearly associated with age, while centromedial amygdala volumes, comprised of the central and medial nuclei, had no relationship with age (Aghamohammadi‐Sereshki et al., 2019). The findings from our study found a positive correlation between all amygdala subnuclei volumes. This suggests growth of the amygdala nuclei during adolescent development display a different relationship than seen in adults.

Future studies should investigate the cellular composition of the amygdala nuclei in neurotypical and atypical populations to determine whether cell types and densities differ between these two groups. Evidence from post‐mortem studies have reported that cellular density and maturation in the various amygdala nuclei differ in children with ASD compared to neurotypical children, and that these groups also differ in nucleic cellular loss and migration during childhood and adolescent development (Avino et al., 2018; Weir et al., 2018). It is currently unknown which type of neurons were involved in these processes.

## 5 CONCLUSIONS

Enlarged BLA nuclei were observed in adolescents with ASD compared to their typically developing peers. Larger BLA and cortical nuclei volumes positively predicted impaired social behaviors, as measured by the ADI‐R, while medial nucleic volumes were negatively associated with both social and communication impairments on the ADOS‐G. Larger Ce nuclei were associated with increased repetitive behaviors. Findings are supported by recent studies examining cellular differences in amygdala subnuclei revealed through post‐mortem analysis of brain tissue acquired in individuals with ASD (Schumann & Amaral 2006). Further examination into the amygdala nuclei growth trajectories and their compositions will help to illuminate which aberrant processes are driving altered nuclei growth in ASD, and how these processes detrimentally affect social behaviors. Findings are supported by recent studies examining cellular differences in amygdala subnuclei revealed through post‐mortem analysis of brain tissue acquired in individuals with ASD (Schumann & Amaral 2006).

Of note is that we did not have access to the information concerning the types of psychiatric disorders typically developing children were excluded for nor were information available as to whether the children with ASD were screened for comorbid disorders at the different sites. As previously introduced, anxiety is a common symptom of ASD, with the comorbidity of ASD and anxiety estimated to be between 55% and 84%. Evidence suggests that anxiety is associated with whole amygdala volumes (Hessl et al., 2021; Juranek et al., 2006). In a study by Herrington et al. (2017), children with ASD with anxiety displayed smaller right amygdala volumes when compared to both TD children and children with ASD who did not have anxiety. No studies have yet examined the relationship between ASD and anxiety on particular subnuclei. Similarly, ASD shares some common features with other disorders, such as OCD and ADHD. It was outside the scope of this project to examine these shared behaviors. Future longitudinal studies with larger sample are needed to examine the development of the amygdala subnuclei from childhood through adolescence. Such studies would benefit from collecting information regarding the pubertal status of all participants to identify the relationships between puberty, amygdala growth, and ASD symptomatology. While the analyses were adjusted for sex, the sample was predominantly right‐handed males, and in turn this may limit the generalizability of the results. For the current study, the ADOS‐G and ADI‐R were used to assess ASD symptoms. While these tools are the gold standard as a diagnostic tool, other measures such as the Social Responsive Scale (Constantino & Gruber 2005) to measure social performance, the Short Sensory Profile that evaluates sensory processing patterns (McIntosh et al., 1999), and the Repetitive Behavior Scale‐Revised (RBS‐R) (Bodfish et al., 2000) to assess restrictive and repetitive behaviors and interests could also be used in conjunction with the diagnostics. A wider assessment of behaviors will aid in identifying which amygdala nuclei contribute to changes in ASD symptomatology across development.

Further examination into the amygdala nuclei growth trajectories and their compositions will help to illuminate which aberrant processes are driving altered nuclei growth in ASD, and how these processes detrimentally affect social behaviors.

## FUNDING INFORMATION

This research was funded by Autism Speaks 04593, K01 NIMH MH081191, NIMH MH67924, NIH HD55748, NICHD P50 HD055784, and NIMH 1R01 HD065280‐01.
